# Impact of Novel Incorporation of CT-based Segment Mapping into a Conjugated Gradient Algorithm on Bone SPECT Imaging: Fundamental Characteristics of a Context-specific Reconstruction Method

**DOI:** 10.22038/AOJNMB.2018.31711.1219

**Published:** 2019

**Authors:** Kyohei Okuda, Susumu Fujii, Shota Sakimoto

**Affiliations:** Department of Clinical Radiology, Tottori University Hospital, Tottori, Japan

**Keywords:** OSCGM, SPECT/CT, Zone-map, ^99m^Tc-MDP

## Abstract

**Objective(s):**

The latest single-photon emission computed tomography (SPECT)/computed tomography (CT) reconstruction system, referred to as xSPECT Bone™, is a context-specific reconstruction system utilizing tissue segmentation information from CT data, which is called a zone map. The aim of this study was to evaluate the effects of zone-map enhancement incorporated into the ordered-subset conjugated gradient minimization (OSCGM) reconstruction method on SPECT images.

**Methods:**

Image quality with zone-map enhanced OSCGM (OSCGMz) and non-enhanced OSCGM methods was compared using various reconstruction parameters. The compartment phantom had 3 radioactive sections with CT values of about 1000, 250, and 0 HU. SPECT data were acquired using a low-energy high-resolution (LEHR) collimator, with a 256×256 matrix and 2.4-mm pixel size. The performances of the 2 reconstruction methods (OSCGM vs. OSCGMz) were evaluated on the basis of %error, coefficient of variation (%CV), and normalized mean squared error (NMSE), and adequate iterative update numbers were determined. The relative CV representing the ratio of smoothed images to non-smoothed images was calculated to evaluate the effects of the Gaussian filter on each section set with different CT values.

**Results:**

On comparing the OSCGM and OSCGMz methods, it was found that the %error of the OSCGMz method tended to show convergence with fewer updates, especially in the high CT value section mimicking bone absorption. In the water section, the %CV of the OSCGMz method was lower than that of the OSCGM method. The NMSE minimum values for the OSCGM and OSCGMz methods were obtained at 30 and 20 updates, respectively. The relative CV for the OSCGMz method in the water section decreased remarkably according to the size of the full width at half maximum (FWHM) of the Gaussian filter.

**Conclusion:**

The zone-map enhancement contributed to SPECT reconstruction for the reproduction of radioactive concentrations in bone tissues, using a low number of OSCGM updates. Our findings indicated that the incorporation of zone maps into SPECT reconstruction might improve image quality.

## Introduction

Computed tomography (CT) has brought about significant advances in single-photon emission computed tomography (SPECT) imaging by enabling the localization of radioactive accumulation through fused images and through compensation of photon attenuation with attenuation maps (1, 2). SPECT/CT fused images facilitate the detection of metastatic lesions or sentinel lymph noodles (3-5), and they allow the distinction of regions showing viability or metabolic capacity (2, 6). Attenuation correction is essential for accurate reproduction of radioactivity distribution in order to perform quantitative assessments using SPECT (7-11). Misregistration between SPECT and CT has reduced because of the widespread use of hybrid SPECT/CT devices. Hence, image degradation factors in SPECT may be more accurately compensated using corresponding CT images.

The latest SPECT/CT reconstruction system being suitable for ^99m^Tc diphosphonate (DPD) SPECT, which is known as xSPECT Bone™ (Siemens Medical Solutions USA Inc., Hoffman Estates, IL, USA), is a context-specific reconstruction system that utilizes tissue segmentation information from CT data (12, 13). In the reconstruction process, the CT data are transformed to zone maps consisting of the following 5 tissue classes: air, fat tissue, soft tissue, medullary bone, and cortical bone. The incorporation of zone maps into SPECT reconstruction using the ordered-subset conjugated gradient minimization (OSCGM) method has remarkably improved bone anatomical appearance in SPECT images. As a clinical application of reconstruction using the zone map-enhanced OSCGM (OSCGMz) method, Kuji et al. (14) reported the utility of skeletal standardized uptake values (SUVs) in patients with prostate cancer. However, fundamental investigations of image quality or optimal imaging parameters with the OSCGMz method have not been performed. Previous studies have reported the characteristics of the OSCGM method (15), including faster convergence of pixel values and divergence of noise when compared to the findings with the ordered-subset expectation maximization (OSEM) method. A novel segmentation algorithm involving zone maps that feature zonal weighting adapted for bone accumulation of radiopharmaceutical agents and separate post-smoothing for each zone may affect these OSCGM characteristics. Additionally, the appropriate imaging parameters may differ between the OSCGMz and the OSCGM methods.

The aim of this study was to evaluate the effects of zone-map enhancement on SPECT images. We first investigated the optimal number of updates in zone map-based OSCGM reconstruction. Subsequently, we investigated the effects on image uniformity at each zone, with the consideration of post-smoothing.

## Methods

***Phantom***

**Figure 1:**
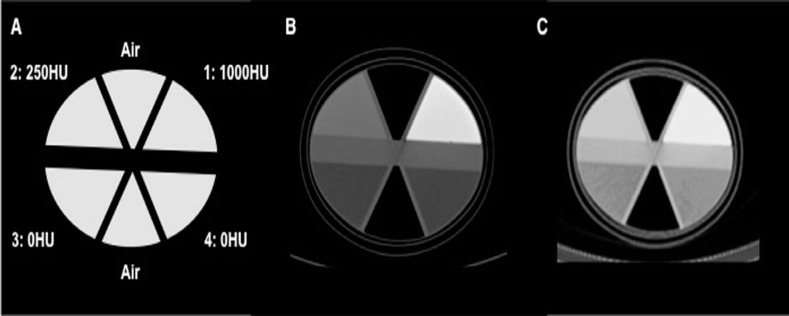
The compartment phantom; schematic structure (A), computed tomography image (B) and corresponding zone-map image (C). The phantom consists of six separate sections, each of which contains materials with different CT values (1000 HU (1), 250 HU (2), 0 HU (3, 4) and air). The zone-map image shows that sections with different CT values are processed as separate segments in the iterative reconstruction

Dipotassium hydrogen phosphate (K_2_HPO_4_) solution (Wako Pure Chemical Industries, Ltd., Osaka, Japan) was used to simulate zonal mapping and absorption by bone tissue. A compartment phantom experiment using the IB-10 brain phantom (Kyoto Kagaku Co. Ltd., Kyoto, Japan) was performed, and the phantom consisted of a non-radioactive water section and 3 radioactive sections supplemented with 80.4 kBq/ml of ^99m^Tc solution and various concentrations of K_2_HPO_4_ vs. pure water. The dose calibrator IGC-7E (Hitachi Aloka Medical Ltd., Tokyo, Japan) was used for radioactive measurement. The CT images and solution contents of the compartment phantom are shown in Figure 1 and Table 1. H-1000 refers to the radioactive section with a CT value of about 1000 HU. Similarly, H-250 and H-0 refer to radioactive sections with CT values of about 250 and 0 HU, respectively. C-0 is the non-radioactive section containing pure water only. The contrast observed in the zone-map image represents the difference in the level of each segment, which is utilized in the OSCGMz method as context-specific information.

***Data acquisition and reconstruction***

The compartment phantom was scanned using the SPECT/CT system (Symbia Intevo; Siemens Medical Solutions USA Inc., Hoffman Estates, IL, USA) equipped with a low-energy high-resolution (LEHR) collimator. The parameters for SPECT acquisition were as follows: continuous acquisition mode with 12 s per projection, 90 projections over a 360° circular orbit, 260-mm rotation radius, 256×256 matrix, and 2.4-mm pixel size. The energy for ^99m^Tc was set in the photopeak window of 129.5–150.5 keV and the scatter window of 108.5–129.5 keV CT was performed at 130 kV and 50 mA in accordance with SPECT acquisition. CT data were subsequently reconstructed with a slice thickness of 2.0 mm and a display field-of-view (DFOV) of 500 mm.

To investigate the effects of the zone map involving the convergence of iterative reconstruction, the acquired projection data were reconstructed with varying numbers of updates ranging from 1 to 90, using the following 2 methods: zone-map enhanced and non-enhanced OSCGM. The subset of OSCGM was fixed at 1, as larger subsets can lead to degradation of image uniformity (15). The Gaussian filter was used for post-smoothing, with various settings of full width at half maximum (FWHM) within 10 mm. Energy window-based scatter correction, CT-based attenuation correction, and resolution compensation with the point spread response function were incorporated in the reconstruction process. All the data were recorded in Bq/ml using the calibration factor measured with the ^99m^Tc point source, as system sensitivity to dose calibrator measurements (1).

***Data analysis***

We first investigated the impact of zone-map enhancement on the number of OSCGM updates by comparing the performances of the 2 reconstruction methods (OSCGM vs. OSCGMz) on the basis of percentage error (%error), coefficient of variation (%CV), and normalized mean squared error (NMSE). In this investigation, the FWHM of the Gaussian filter was fixed at 5 mm. Circular regions of interest (ROIs) in each section were drawn on 5 consecutive slices in the center of the compartment phantom in order to determine the %error and %CV. Indices relevant to the effects of the zone map were calculated as follows:

%error=MEANiACTIVITY-1×100

%CV=SDiMEANi×100

where MEAN_i_ represents the mean SPECT values (kBq/ml) in the ROIs for radioactive section i, ACTIVITY represents the radioactivity of 80.4 kBq/ml contained in the 3 radioactive sections, and SD_i_ represents the standard deviation of the ROIs for radioactive section i.

NMSE=∑i=1x∑j=1y∑k=1zRi,j,k-Ti,j,k2∑i=1x∑j=1y∑k=1zRi,j,k2

where R(*i, j, k*) and T(*i, j, k*) represent a reference standard image and a test image, respectively. The acquisition time for the reference image was set at 120 s per projection corresponding to 10 times that of the test image.

Subsequently, the effects of post-smoothing on each section set with different CT values were evaluated by varying the FWHM of the Gaussian filter. We compared the post-smoothing effects using the 2 reconstruction methods, and each of these used adequate updates as determined by the NMSE results. The index representing the post-smoothing effects for image uniformity was the relative CV, and it was calculated as follows:

RelativeCV=CVT,iCVR,i

where CV_T,i_ represents the CV (%) in the ROIs for radioactive section i in the test image with smoothing, while CV_R,i_ represents the CV (%) in the ROIs for radioactive section i in the reference image without smoothing.

Data analyses were performed using OsiriX DICOM viewer version 5.6 (Pixmeo, Geneva, Switzerland).

## Results

**Figure 2:**
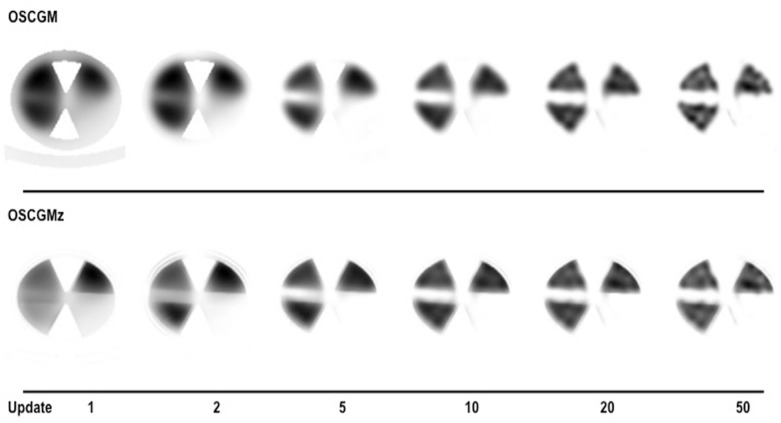
OSCGM and OSCGMz reconstructed images are shown with varied update numbers. The FWHM of the Gaussian filter is fixed at 5 mm. The clear boundaries of the OSCGMz reconstructed images are different from those in conventional blurred SPECT images, and rather resemble the boundaries in CT images. Boundaries with air may be found more easily owing to the imaging model assuming that a radionuclide cannot accumulate on air

**Figure 3:**
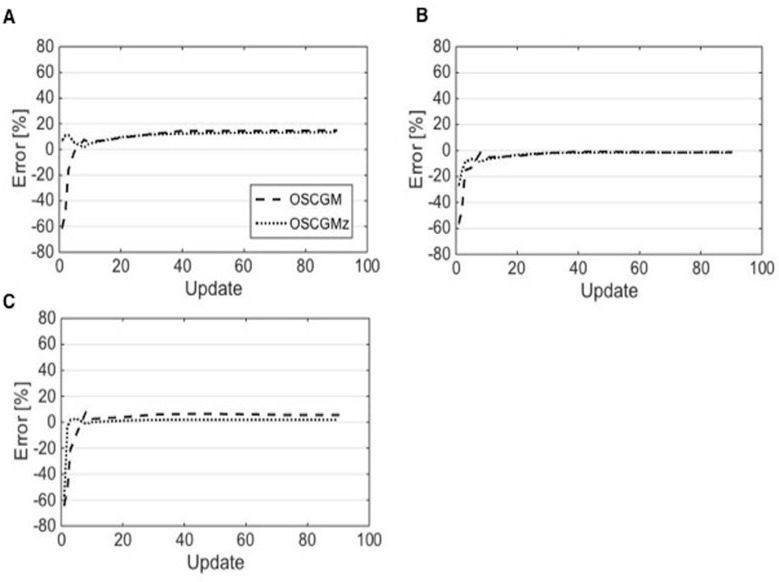
The %error for the OSCGM and OSCGMz methods plotted as a function of the update number in sections H-1000 (A), H-250 (B), and H-0 (C). Convergence of %error for the OSCGMz method is observed with very few updates. This tendency is notable in the high CT value section

**Figure 4:**
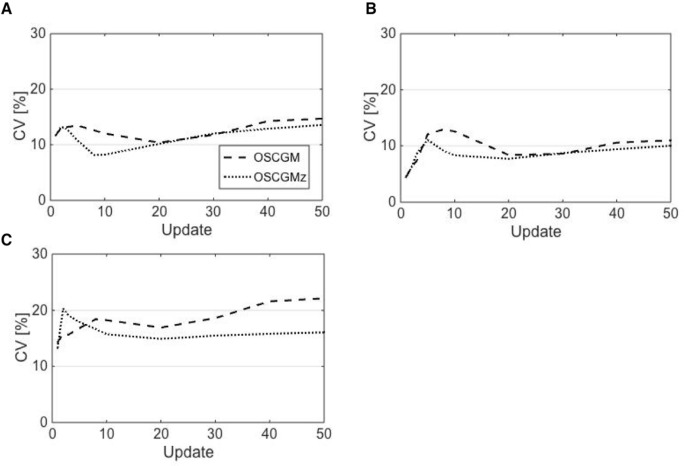
The %CV for the OSCGM and OSCGMz methods plotted as a function of the update number in sections H-1000 (A), H-250 (B), and H-0 (C). In the H-0 section, the %CV for the OSCGMz method was stable at over 10 updates, while that for the OSCGM method continuously increased as the update number increased

Both the OSCGM and OSCGMz methods yielded images that were distinct from conventional SPECT images, although the images with the latter method appeared to be closer to CT images (Figure 2). The OSCGM method with 1 update showed a typical image representative of a novel feature in the reconstruction process, assuming that the presence of radioactivity was unlikely in the air region restricted by CT values. Both of the methods appeared to achieve reproduction of the radioactive concentration in the compartment phantom through reconstruction with update numbers of 20 or larger. Figure 3 presents the %errors obtained for all 3 radioactive sections. On comparing the OSCGM and OSCGMz methods, it was found that the %error of the OSCGMz method tended to show convergence with fewer updates. This tendency was noticeable in the higher CT value sections. Convergence of the %error with the OSCGMz method was achieved with very few updates, while that with the OSCGM method necessitated over 10 updates. A difference in the %error between the reconstruction methods was not observed when 10 or more updates were considered. The %CV results of all 3 radioactive sections are shown in Figure 4. Negligible differences in the %CV were observed between the 2 methods with regard to the H-1000 and H-250 sections, when the number of updates with the OSCGM method was over 20. However, in the H-0 section, the %CV was lower with the OSCGMz method than with the OSCGM method, when a large number of updates were used. 

**Figure 5:**
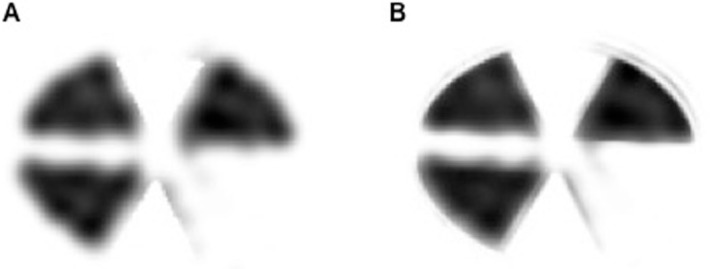
Reconstructed images of the compartment phantom, with equivalent quantitative accuracy and image uniformity. The OSCGM method involved 30 updates and an 8-mm FWHM of the Gaussian filter (A), while the OSCGMz method involved 20 updates and a 4-mm FWHM of the Gaussian filter (B)

The NMSE minimum values for the OSCGM and OSCGMz methods were obtained at 30 and 20 updates, respectively. On the basis of these results, we determined the adequate number of updates for the OSCGM and OSCGMz methods as 30 and 20, respectively. The %error and %CV for the 2 methods using this adequate number of updates are summarized in Table 2. The differences in the %error in each section among the tested parameter settings were within 10%. The %CV was lower with the OSCGMz method than with the OSCGM method, and there was no need for a large FWHM to achieve post-smoothing. The quantitative accuracy and uniformity of reconstructed images were equivalent between the OSCGMz method using 20 updates and a 4-mm FWHM of the Gaussian filter and the OSCGM method using 30 updates and an 8-mm FWHM of the Gaussian filter (Figure 5).

**Figure 6:**
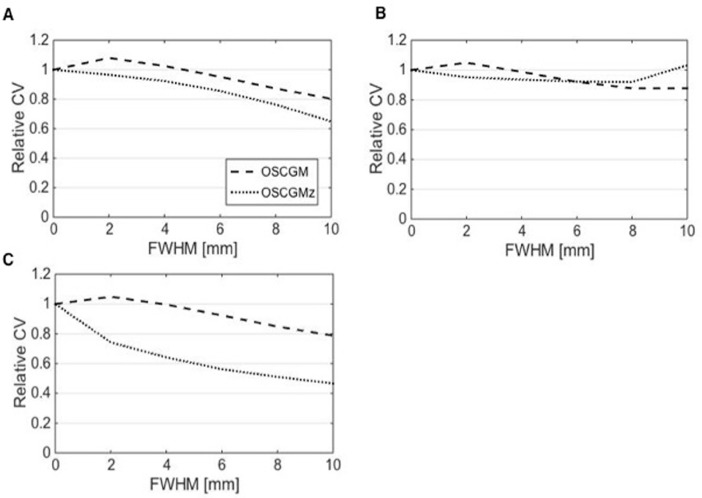
Relative CV for the OSCGM and OSCGMz methods indicates the effects of post-smoothing against non-smoothing in sections H-1000 (A), H-250 (B), and H-0 (C). The relative CV for the OSCGMz method in the H-0 section is greatly decreased according to the FWHM size, and the effect of post-smoothing is distinctive

The results of the relative CV are shown in Figure 6 In all the radioactive sections, the relative CV for the OSCGM method showed a slight decrease according to the size of the FWHM of the Gaussian filter. The relative CV with the OSCGMz method showed a tendency to resemble that with the OSCGM method, except in the H-0 section. The post-smoothing effect for the OSCGMz method in the H-0 section was greater than that for any other tested section.

## Discussion

Novel technologies as zone maps derived from CT data can be utilized to obtain contextual information for adapting to accumulation of ^99m^Tc DPD, and they can enhance OSCGM reconstruction for bone SPECT (13). In this study, we investigated the effects of zone-map enhancement on OSCGM reconstruction. Our results using a simple compartment phantom suggested that the incorporation of zone maps into OSCGM reconstruction affects not only the appearance of SPECT images, but also imaging parameters, such as the OSCGM update number and post-smoothing intensity.

**Table 1:** Solution content within each section of the compartment phantom

**Section**		^99m^ **Tc solution ** **(kBq/ml)**	**CT value (HU)**
1	H-1000	80.4	1000
2	H-250	80.4	250
3	H-0	80.4	0
4	C-0	0	0

Three sections have computed tomography values (1000, 250, and 0 HU) mimicking bone absorption and one includes water

**Table 2:** Comparisons of %error and %CV between the OSCGM and OSCGMz methods with adequate update numbers

		**Error (%)**			**CV (%)**		
**Methods**	**Gaussian filter** **FWHM (mm)**	**H-1000**	**H-250**	**H-0**	**H-1000**	**H-250**	**H-0**
OSCGM update 30	2	14.87	0.23	7.99	14.02	9.96	21.42
	4	13.58	-0.87	6.78	13.31	9.37	20.34
	8	9.31	-4.62	2.82	11.33	8.33	17.32
OSCGMz update 20	2	10.37	-2.54	5.36	10.83	7.69	18.50
	4	9.60	-3.30	2.44	10.35	7.56	15.60
	8	7.37	-5.65	-3.47	8.56	7.43	12.72

Quantitative accuracy and image uniformity are equivalent between the OSCGM and OSCGMz methods, when the FWHM values of the Gaussian filter are 8 mm and 4 mm, respectively

The %error of the OSCGMz method tended to converge with fewer updates when compared to the number of updates required with the OSCGM method. The optimal number of updates was lower for the OSCGMz method than for the OSCGM method (20 and 30, respectively), according to the NMSE results. Hence, zone-map enhancement contributed to the convergence of the radioactive concentration on OSCGM reconstruction, and it may improve image uniformity, which classically degrades with an increase in the number of updates during the process of iterative reconstruction (16, 17). Our results showed also a remarkably faster convergence for the OSCGMz method in the section imitating bone equivalent density at a higher concentration of K_2_HPO_4_. Cachovan et al. (18) reported a significant correlation between ^99m^Tc DPD uptake and the CT value. Accumulation of ^99m^Tc DPD is probably in the region with a higher CT value; therefore, zone-map enhancement helps estimate the relative amount of uptake during the iterative reconstruction. As such, incorporating the probability of ^99m^Tc uptake into image reconstruction may be considered as the main approach for faster convergence in bone equivalent sections.

In contrast to faster convergence in bone equivalent sections, improvement in image uniformity on zone-map enhancement was clear in the section filled with pure water. The post-smoothing effect on zone-map enhancement, as shown by the relative CV results, was also distinct in the water section. The incorporation of zone maps into SPECT reconstruction allows separate processing of each zone restricted according to the CT value. In xSPECT Bone, each zone is separately smoothed out with a 3D Gaussian filter having a FWHM for bone zones that is half the value for non-bone zones (13). As shown in our study, zone-map enhancement may improve image uniformity remarkably in non-bone zones that have low radioactivity accumulation, and hence, an increase in image noise might be assumed. In brief, the OSCGMz method helps improve image uniformity; hence, a large FWHM of the Gaussian filter might not be needed. As the trade-off between image uniformity and contrast is controlled by the FWHM of the Gaussian filter, the novel zone-mapping approach might help mitigate the degradation of image resolution by post-smoothing. The characteristics of the OSCGMz method, including faster iterative convergence, improvement in image uniformity, and boundary preservation, were suggested in the present study, and these provide images that are distinct from conventional blurred SPECT images.

With regard to the quantitative results, the OSCGMz and OSCGM methods had few differences under the condition of adequate updates (2–5%). Among all the sections, the largest error was observed in the H-1000 section. This result may be explained by errors occurring during the preparation of the solution in the H-1000 section, including a high density of K_2_HPO_4_. However, the most important observation in this study is that zone-map enhancement does not affect the quantitative voxels reconstructed with the OSCGM method.

To explain the effect of zone-map enhancement on OSCGM reconstruction, we used a simple compartment phantom, and we identified the adequate update number with the OSCGMz method as 20. Thus, more optimal imaging parameters in the OSCGMz method should be explored with a torso phantom mimicking bone. A SPECT image is inferior with regard to image resolution when compared to positron emission tomography (PET), CT, and magnetic resonance imaging (MRI) images. The partial volume effect is an inevitable issue in the degradation of image resolution (19), and it should be taken into account in quantitative assessment (20). Recently, the SUV in SPECT has been discussed thoroughly with the aim of utilizing this indicator for differential diagnosis or assessment of the therapeutic effect (21-23). In the present study, the effect of zone-map enhancement on partial volume in SPECT images was not evaluated. It is interesting to determine whether zone maps can recover the poor resolution of SPECT images.

## Conclusion

Using a compartment phantom, we revealed the effects of novel zone mapping on SPECT images. Zone-map enhancement contributed to SPECT reconstruction for the reproduction of radioactive concentrations in bones, using a low number of OSCGM updates. Image uniformity in non-bone regions might be improved greatly by the incorporation of this algorithm into SPECT reconstruction. Our study suggested that zone maps influence image quality as well as the appearance of reconstructed images. Further examinations are necessary to optimize quantitative bone SPECT.
